# High-quality III-nitride films on conductive, transparent (2̅01)-oriented β-Ga_2_O_3_ using a GaN buffer layer

**DOI:** 10.1038/srep29747

**Published:** 2016-07-14

**Authors:** M. M. Muhammed, M. A. Roldan, Y. Yamashita, S.-L. Sahonta, I. A. Ajia, K. Iizuka, A. Kuramata, C. J. Humphreys, I. S. Roqan

**Affiliations:** 1 King Abdullah University of Science and Technology (KAUST), Physical Sciences and Engineering Division, Thuwal 23955-6900, Saudi Arabia; 2 Imaging and Characterization Core Lab, King Abdullah University of Science and Technology (KAUST), Thuwal 23955-6900, Saudi Arabia; 3 Tamura Corporation, Sayama, Saitama 350-1328, Japan; 4 Department of Materials Science and Metallurgy, University of Cambridge, 27 Charles Babbage Road, Cambridge CB3 0FS, United Kingdom

## Abstract

We demonstrate the high structural and optical properties of In_x_Ga_1−x_N epilayers (0 ≤ x ≤ 23) grown on conductive and transparent (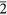
01)-oriented β-Ga_2_O_3_ substrates using a low-temperature GaN buffer layer rather than AlN buffer layer, which enhances the quality and stability of the crystals compared to those grown on (100)-oriented β-Ga_2_O_3_. Raman maps show that the 2″ wafer is relaxed and uniform. Transmission electron microscopy (TEM) reveals that the dislocation density reduces considerably (~4.8 × 10^7^ cm^−2^) at the grain centers. High-resolution TEM analysis demonstrates that most dislocations emerge at an angle with respect to the *c*-axis, whereas dislocations of the opposite phase form a loop and annihilate each other. The dislocation behavior is due to irregular (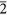
01) β-Ga_2_O_3_ surface at the interface and distorted buffer layer, followed by relaxed GaN epilayer. Photoluminescence results confirm high optical quality and time-resolved spectroscopy shows that the recombination is governed by bound excitons. We find that a low root-mean-square average (≤1.5 nm) of In_x_Ga_1−x_N epilayers can be achieved with high optical quality of In_x_Ga_1−x_N epilayers. We reveal that (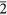
01)-oriented β-Ga_2_O_3_ substrate has a strong potential for use in large-scale high-quality vertical light emitting device design.

Wide bandgap III-nitride semiconductors have several material properties that make them attractive for potential application to devices that emit and detect light in the spectrum between ultraviolet (UV) and visible light and for high-power electronic devices^1^,^2^,^3^. Therefore, both research and industry are motivated to produce high-performing III-nitride devices. However, the high cost of native substrates is one of the key challenges to overcome in the field, and commonly used substrates (sapphire (Al_2_O_3_), SiC, or Si) add complexity and cost to the device fabrication. Particularly, III-nitride based vertical devices, such as vertical-cavity surface-emitting lasers (VCSELs), are costly and challenging to produce, thus compromising on the efficiency obtained. For example, the efficiency of devices grown on Al_2_O_3_ is threatened by the introduction of high threading dislocation density (TDD)^4^,^5^ due to a large lattice mismatch (14%) and a large difference in thermal expansion coefficients between III-nitride semiconductor and the substrate^6^,^7^. This high TDD causes many nonradiative recombinations and scattering centers, which will deteriorate the optical and electrical quality of III-nitride devices^8^. To date, several attempts have been made to increase the quantum efficiency, albeit with limited success. For example, although threading dislocations (TDs) ending with V-shaped defects in InGaN/GaN light-emitting diodes (LEDs) help to reduce the nonradiative recombination inside TDs, the total effective area contributing to the emission is still reduced^9^. Furthermore, high TDD leads to efficiency decline in AlGaN/GaN UV LEDs^10^. Therefore, low TDD is preferable, as it can improve the continuous operation lifetime for devices and high quantum efficiency^8^. Moreover, as Al_2_O_3_ substrates are insulating, the contacts should be established at the top surface of the devices. This not only reduces the total available light emission area but also increases the fabrication process complexity, in addition to requiring specialized packaging and integration of devices. Moreover, LEDs grown on Al_2_O_3_ exhibit current crowding due to lateral injection, which leads to poor current management and heat distribution, undermining device efficiency. For devices grown on SiC, micropipes are introduced during crystal growth, preventing the use of full wafers. In addition, SiC is very expensive and the incorporation of N_2_ dopants diminishes its transparency^11^. Even though this substrate is characterized by a smaller lattice mismatch (3.1%)^12^ compared to Al_2_O_3_ and provides vertical current injection geometry, the lack of the transparency of this conductive SiC decreases the LED efficiency due to the substrate light absorption. Therefore, as additional processing steps are required, the device fabrication is made more complex. Efforts are being made to enhance the light efficiency of LEDs on SiC by using substrate transferring technique (substrate liftoff or introduction of distributed Bragg reflectors (DBRs) between the nitride and the substrate to reflect the light back from the substrate are employed)^13^. Nonetheless, producing good quality nitride DBRs remains a challenge. Therefore, there is still a significant need for alternative substrates that can be employed in the fabrication of bright vertical light-emitting devices with good lattice match, high thermal and electrical conductivity, and high transparency in UV spectral regions.

The β-Ga_2_O_3_ substrate combines the beneficial properties (low lattice mismatch, transparency and conductivity) of both Al_2_O_3_ and SiC. β-Ga_2_O_3_ is a promising candidate as a wide bandgap (4.8 eV)^14^ substrate for fabricating tunable bright III-nitride vertical light-emitting devices because it satisfies these conditions and surpasses several other substrates. Conducting β-Ga_2_O_3_ allows vertical current flow, reduces the forward operating voltage and series resistance, and improves current distribution and thermal management. Conductive substrates allow fabricating the contacts at both the top and the bottom surface, which simplifies device fabrication, integration process, and packaging. This approach also reduces the fabrication cost and increases the number of devices in a single chip, resulting in greater light extraction efficiency (LEE) compared to Al_2_O_3_ substrates. Furthermore, the transparent nature of β-Ga_2_O_3_ as a substrate provides a wider light-emitting area than do conductive SiC and Si substrates. Therefore, emission from vertical devices grown on β-Ga_2_O_3_ is omnidirectional, which further increases the LEE, resulting in a bright-light-emitting device and supporting high power operations. Conversely, emission from that grown on SiC is permitted through the top side only. In addition, Ga_2_O_3_ shows high stability at high growth temperatures of around 1100 °C^15^. Relative to GaN substrates, Ga_2_O_3_ has a wider bandgap and is cheaper to grow and process.

Previous attempts to grow high-quality III-nitride quantum-well-based (100)-oriented β-Ga_2_O_3_ have been unsuccessful, as the quality of these crystals is inadequate for high-performance devices^16^,^17^,^18^. The strong cleavage nature of the (100)-plane caused the GaN epilayer to detach and peel off from the (100) β-Ga_2_O_3_ plane^19^, causing complications to the required step of separating wafers by dicing. In our previous study, we found that using monoclinic (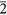
01)-oriented β-Ga_2_O_3_ substrate led to a high optical and structural quality GaN material using an AlN buffer layer^20^. Using this (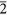
01)-oriented β-Ga_2_O_3_ substrate, we found that (0002) GaN rocking curve (RC) has a significantly enhanced full width at half maximum (FWHM) value (430 arcsec)^20^ compared to that grown on (100)-oriented β-Ga_2_O_3_ (1200 arcsec)^21^. We reported a relatively low lattice mismatch of ~4.7% and an in-plane epitaxial orientation relationship of (010) β-Ga_2_O_3_ || (11
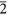
0) GaN and (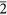
01) β-Ga_2_O_3_ || (0001) GaN^20^. The growth of InGaN LEDs on (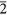
01) β-Ga_2_O_3_ with an AlN buffer layer is presently under investigation^22^.

In this paper, we demonstrate high optical and structural quality of Si-doped GaN and In_x_Ga_1−x_N epilayers grown on (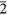
01) β-Ga_2_O_3_ substrate using a low-temperature GaN buffer layer by metal organic chemical vapor deposition (MOCVD). Our results are a testament to the potential for producing InGaN vertical LEDs that are more efficient, of better quality, and more simply and cheaply produced than LEDs grown on Al_2_O_3_ or SiC.

## Growth and Characterization

The low-temperature undoped GaN buffer layer was grown to a thickness of ~9 nm at 500 °C under an N_2_ and NH_3_ atmosphere on (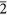
01)-oriented monoclinic conductive β-Ga_2_O_3_ substrates using a low-pressure, vertical MOCVD reactor. The (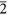
01) β-Ga_2_O_3_ substrate was doped with Sn to increase its conductivity, whereby Hall measurements revealed an electron concentration in the order of 10^18^ cm^−3^. A low-temperature grown GaN buffer layer is known to decrease hillocks in the GaN epilayer surface^23^,^24^,^25^ on the Ga_2_O_3_ substrate and decrease the TDD compared to that on AlN buffer layer^20^. After changing the carrier gas from N_2_ to H_2_, the temperature of the substrate was increased to 1020 °C to grow the Si-doped GaN (n-GaN) epilayer (with a carrier density of 4 × 10^18^ cm^−3^ and a nominal thickness of ~1.75 μm). The temperature was further increased to 1100 °C during the deposition of the remainder of the Si-doped GaN layer (of ~1.75 μm thickness). This two-step growth process decreases the formation of epicracks by reducing dislocations between the GaN layers grown in the first and the second steps^26^,^27^. Current-voltage measurements confirmed that the interface between the Ga_2_O_3_ substrate and the GaN epilayer is conductive. Transmission electron microscopy (TEM) images confirmed that the total thickness of the GaN epilayer was 3.7 μm. In_x_Ga_1−x_N layers (5 ≤ x ≤ 23) with a nominal thickness of 40 nm were grown on n-GaN/Ga_2_O_3_ with a GaN buffer layer by MOCVD. During the In_x_Ga_1−x_N layer growth, precursors of trimethylgallium (TMGa), trimethylindium (TMIn), and NH_3_ were used as source gasses and N_2_ as the carrier gas at a pressure 400 mbar. The growth temperature was varied for different x values: 0.01 (875 °C), 0.05 (820 °C), 0.1 (795 °C), 0.15 (775 °C), and 0.23 (720 °C). For the purpose of comparison, we grew In_x_Ga_1−x_N films on Al_2_O_3_ using similar optimization growth process.

Cross-sectional and plan-view TEM specimens were prepared using a lamellar lift-out procedure on an FEI Helios focused ion beam scanning electron microscope, and cross-sectional imaging was performed on a JEOL 4000 EX TEM operating at 400 kV. The plan-view TEM images were obtained using FEI Tecnai TWIN TEM operated at 120 kV. Atomic resolution High Angle Annular Dark Field-Scanning Transmission electron microscopy (HAADF-STEM) study was carried out with a Titan Cs-Probe Corrected (FEI Co.) microscope operated at 300 kV. In order to get the strain maps from the HAADF images, we used The Geometric Phase Analysis (GPA) plug-in package (HREM Research Inc.), which was implemented in the Digital Micrograph software (Gatan). The surface morphology and roughness of the InGaN epilayers were examined by atomic force microscopy (AFM) using the Agilent 5400 scanning probe microscope. The X-Ray Diffraction (XRD) was performed on a Bruker D8 diffractometer system using a Cu K_α1_ radiation. The emission characteristics of this GaN epilayer were compared to a similar GaN epilayer grown by the same growth process but with an AlN buffer layer. Photoluminescence (PL) was measured to investigate the optical properties of the n-GaN film using a 325-nm He-Cd laser at different temperatures. The spectra were collected by an Andor monochromator attached to a charge-coupled device camera. The samples were mounted in a closed-cycle helium cryostat for low-temperature PL (6 K). Time-resolved PL (TRPL) experiments were carried out with a Hamamatsu Synchroscan streak camera. The samples were excited by the third harmonic UV (λ = 266 nm) pulses of a mode-locked Ti:sapphire femtosecond pulsed laser (frequency was doubled using a barium borate crystal) with a pulse width of ~150 fs and a power density of 70 W/cm^2^ (with 76 MHz repetition rate). A Coherent Verdi-V18 diode-pumped solid-state continuous wave laser was used to pump the Ti:sapphire laser. Emission of the sample was detected by a monochromator attached to a UV-sensitive Hamamatsu C6860 streak camera with a temporal resolution of 2 ps. The samples were mounted in a variable temperature open-helium cryostat for measurements between 2 and 300 K.

## Results and Discussion

### Structural characterization of GaN 2″ wafer

**Figure 1:**
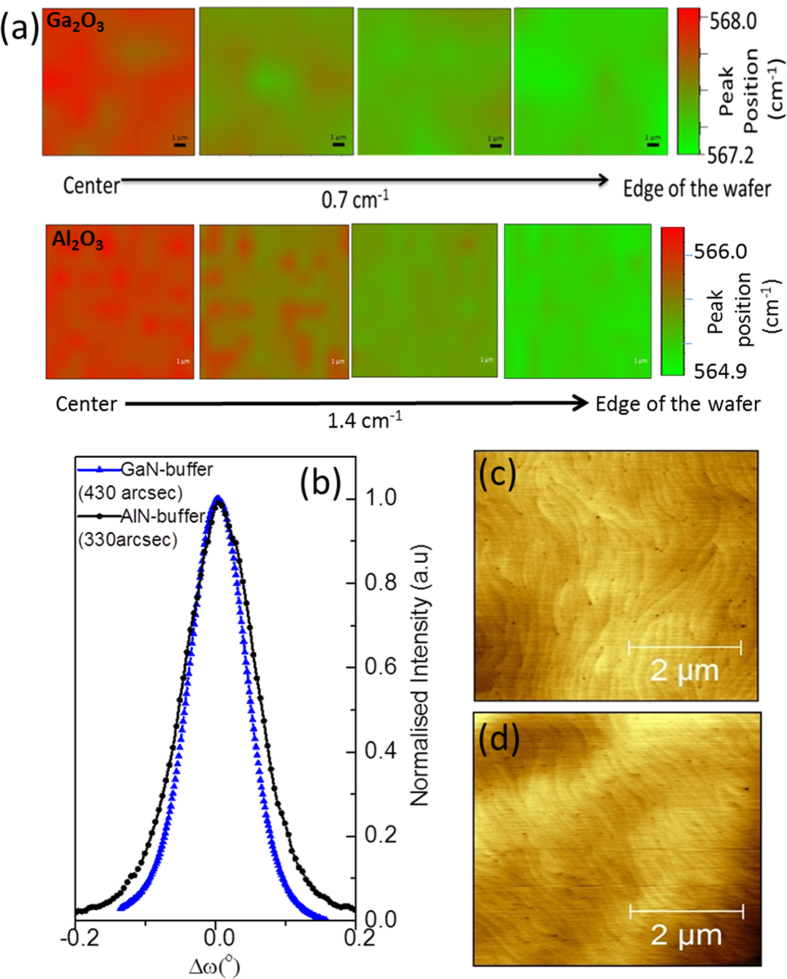
(**a**) Raman mapping of the E_2_(high) peak position from the center (*red*) to the edge (*green*) of the 2″ n-GaN/(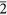
01)-oriented β-Ga_2_O_3_ wafer (top panel) and n-GaN/sapphire wafer (bottom panel). (**b**) The XRD RC around GaN (0002) reflection peak on n-GaN/(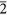
01)-oriented β-Ga_2_O_3_ wafers with GaN buffer (FWHM = 330 arcsec) and AlN buffer (FWHM = 430 arcsec). 5 × 5-μm^2^ AFM image of the same wafers with (**c**) GaN as the buffer layer and (**d**) AlN buffer layer.

It is important to obtain high quality and uniform wafers for large-size fabrication technology for use in vertical emitting devices. Raman spectroscopy and XRD measurements were used to examine the material quality and uniformity across the whole 2″ wafer. The strain in GaN/(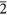
01) β-Ga_2_O_3_ epilayer was estimated by Raman measurements. Figure 1(a) (top panel) indicates the Raman mapping E_2_(high) peak position across the 2′′ GaN/β-Ga_2_O_3_ wafer. The E_2_(high) mode was confirmed to be sensitive to biaxial strain in GaN epilayers^28^. The Raman maps reveal a homogenous uniform stress distribution over the entire wafer with a negligible left shift of ~0.7 cm^−1^ observed between the center (567.3 cm^−1^) and the edge (568.0 cm^−1^) of the wafer, indicating that the stress is fully released to the edge. The E_2_(high) peak exhibits an average value of ~567.93 cm^−1^ with a very small left shift (−0.07 cm^−1^) compared to that of bulk relaxed GaN (568 cm^−1^)^28^. This strain value indicates that the uniform GaN/Ga_2_O_3_ wafer is nearly strain-free, whereby the presence of a very slight tensile strain suggests low TDD (It is noteworthy that the low lattice mismatch between (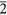
01) β-Ga_2_O_3_ and the GaN film (~4.7%) can effectively reduce TDD). On the other hand, a high-quality GaN/Al_2_O_3_ 2″ wafer (grown with the same structure) shows a considerable left shift of ~1.4 cm^−1^ (Fig. 1(a) bottom panel). Furthermore, GaN grown on AlN buffer layer showed a compressive strain in the film with the E_2_(high) peak shifted to the right by 1.04 cm^−1^ compared to the bulk GaN^20^. The wafer uniformity of high-quality GaN grown on (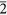
01)-oriented β-Ga_2_O_3_ is enhanced significantly using GaN buffer layer by formation of nucleation centers, which is particularly beneficial for good quality two-dimensional lateral growth GaN layer^29^.

To study the reduction in TD, AFM and XRD measurements were conducted across the wafer. The XRD RC analysis of the GaN (0002) reflection peak was performed on the GaN/(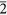
01) β-Ga_2_O_3_ wafer. The FWHM value showed a sharper peak of ~330 arcsec (Fig. 1(b)), disclosing a better quality GaN epilayer grown on GaN buffer layer compared to that grown on AlN buffer layer (~430 arcsec), as shown in Fig. 1(b). To the best of our knowledge, this is the best RC FWHM value for GaN obtained for materials grown on a Ga_2_O_3_ substrate. This FWHM of the RC confirms that (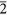
01) orientation of β-Ga_2_O_3_ substrates improves the crystal quality significantly compared to the (100) orientation (1200 arcsec)^21^. The surface morphology of GaN epilayers was analyzed by examining the AFM images across the whole wafer. Figure 1(c) illustrates that the GaN grown on (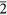
01) β-Ga_2_O_3_ with GaN buffer layer confirms a decrease in TDD (>50%) compared to that with AlN buffer layer (Fig. 1(d)) using the same substrate. The average TDD count (obtained by averaging over >20 AFM 5 × 5 μm^2^ images at different positions on the 2″ wafer) on GaN buffer layer is found to be 1.8 (±0.2) × 10^8^ cm^−2^ and that on AlN buffer is 4.5 (±0.2) × 10^8^ cm^−2^. This low TDD confirms the Raman results, indicating that such substrate can be used for large-scale technology based on GaN vertical light emitting devices.

### TEM analysis and low TDD

**Figure 2:**
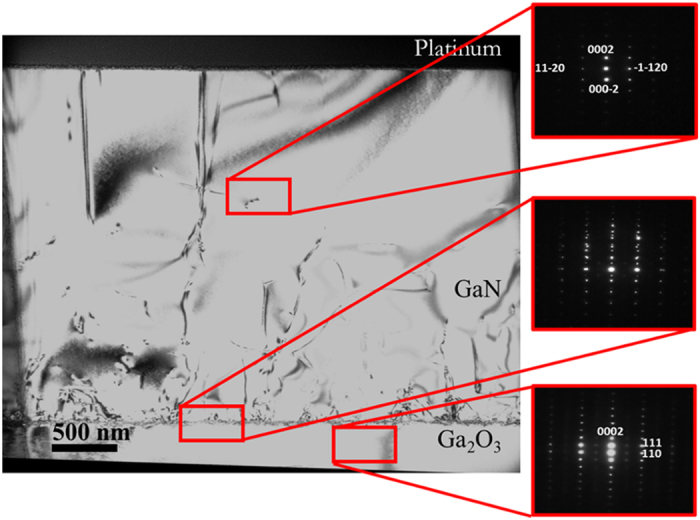
A cross-sectional TEM image of the n-GaN/(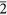 01)-oriented β-Ga_2_O_3_ using a GaN buffer layer with electron diffraction patterns collected from the GaN epilayer (top), interface (middle), and substrate (bottom).

TEM and high-resolution TEM (HRTEM) must be carried out to investigate the actual TDD and analyze the dislocation mechanism. The interface between GaN/β-Ga_2_O_3_ was studied by cross-sectional TEM, as shown in Fig. 2. The samples were examined by diffraction contrast, viewing close to the [1
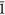
00] zone axis by tilting the sample to excite the *g* = (0002) and (1
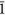
20) Bragg reflections. The electron diffraction pattern taken from the GaN epilayer (the top inset in Fig. 2(a)) shows a single crystal wurtzite (0001) structure with no zinc-blende regions or misoriented grains. Diffraction patterns pertaining to the labeled regions in Fig. 2 are taken near the interface (center inset), revealing a strong epitaxial relationship between the substrate and the GaN epilayer. The bottom inset of Fig. 2 shows an image taken along the GaN [1
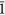
00] zone axis, parallel to the β-Ga_2_O_3_ [110] direction.

**Figure 3:**
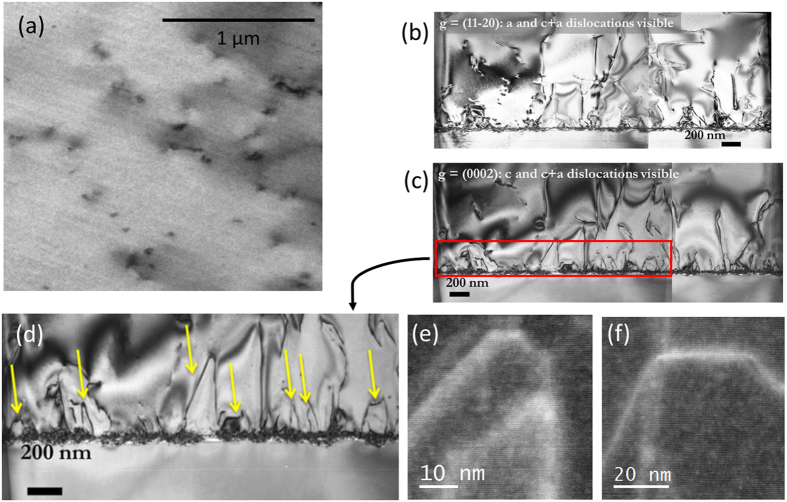
(**a**) Bright-field plan-view TEM micrograph of the n-GaN epilayer showing surface dislocation densities. Cross-sectional TEM image showing a detailed view of the dislocations with (**b**) g = (1
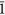
20) and (**c**) g = (0002) imaging conditions. (**d**) A magnified part from cross-sectional view with g = (0002) (panel **c**), indicating the closed dislocation loops pointed by yellow arrows. (**e**,**f**) HRTEM of some of the eliminated dislocation loops near the interface.

Plan-view TEM analysis was performed to confirm the TDD on the epilayer by bright-field imaging in the vicinity of the [0001] zone axis. Figure 3(a) shows a plan-view TEM image taken under two-beam diffraction conditions, whereby *g* = (11
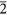
0) to allow for accurate determination of the TD from an average of 40 images. The TDD obtained at the centers of GaN grains (of ~1–~2-μm diameter) is considerably lower (~4.8 × 10^7^ cm^−2^) for that grown on Ga_2_O_3_ than that grown on a flat Al_2_O_3_ using conventional MOCVD^30^. As can be seen in Fig. 3(a), TDs appear to migrate to the grain boundaries and are depicted in the image as chains of dark spots delineating the grain boundary. The total average TDD is found to be low (~1.9 (±0.2) × 10^8^ cm^−2^). This TDD value is in good agreement with the results obtained by Raman and AFM analysis.

### HRTEM analysis and TD annihilation mechanism

To investigate the TDD reduction mechanism, we carried out TEM and HRTEM analyses. The *c*- and *a*-components of the Burgers vectors of the TDs are visible, as shown in the cross-sectional images in Fig. 3(b,c), respectively. With diffraction condition *g* = (0002), only screw and mixed dislocations are visible, whereas edge and mixed components are visible in the *g* = (1
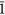
20) condition^31^. The (a):(c+a) dislocation ratio is observed to be approximately 1:2, thus differing from the 1:1 ratio typical for dislocation types in standard low TDD GaN grown on (0001) Al_2_O_3_^32^. The TEM cross-sectional images (Fig. 3(b,c)) for *g* = (0002) and *g* = (1
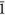
20) show that the GaN/Ga_2_O_3_ interface is abrupt and characterized by a high initial TDD followed by a gradual reduction in the GaN layer (at the distance <200 nm from the substrate) in density as the film continues to grow. Figure 3(d) shows closed dislocation loops (pointed by yellow arrows, taken with g = (0002)). Figure 3(e,f) show a HRTEM cross-sectional view with *g* = (0002) reflections of TD loops. Figure 3(d–f) demonstrate that the TDs bend into the basal plane and react with dislocations of the opposite phase, and are eliminated by forming closed loops in the low-temperature GaN buffer layer and in the lower regions of the GaN epilayer. Therefore, the dislocation does not propagate to the upper part of the GaN epilayer.

**Figure 4:**
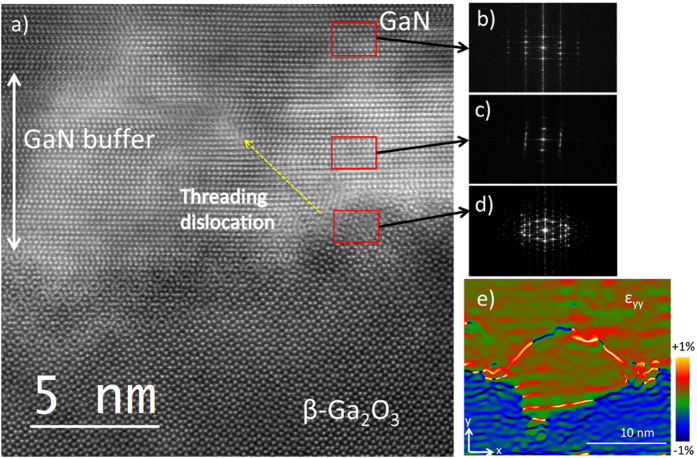
(**a**) HRTEM images of the interface between the overlying GaN layer, low-temperature grown GaN buffer layer, and the (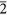
01)-oriented β-Ga_2_O_3_ substrate. An angled Dislocation originating from the interface is shown with a yellow dotted arrow. FFT images are taken from the (**b**) overlaying n-GaN epilayer, (**c**) the GaN buffer layer and the (**d**) interface are shown at different positions (red rectangles). (**e**) Strain map by GPA method on the area shown in (**a**) in y-direction as shown in the figure.

HRTEM and FFT analyses were carried out to investigate the origin of the TD annihilation mechanism. Figure 4(a) shows the HRTEM image of the overlying GaN epilayer, low-temperature grown undoped GaN buffer layer (9 ± 1 nm) and the interface between the buffer layer and the monoclinic β-Ga_2_O_3_ substrate. The surface of the flat β-Ga_2_O_3_ substrate is characterized by slightly irregular surface “nano hump-features” that reach <4 nm height at the interface. As a result of this irregular feature, the dislocation was grown with an angle with respect to the [0001] direction^33^, as indicated by the dotted yellow arrow in Fig. 4(a). These dislocation types usually bend and propagate horizontally^33^. FFT images taken from the overlaying n-GaN epilayer, the buffer layer, and the interface are indicated in Fig. 4(b–d), respectively. FFT image (Fig. 4(d) of the interface reveals a complete distortion. This distortion extends to the buffer layer, as shown in the FFT image (Fig. 4(c)) of the low-temperature GaN buffer layer, followed by highly crystalline roughly relaxed n-GaN epilayer, depicted in the FFT image (Fig. 4b). The distortion of the buffer layer may absorb the effect of misfit dislocations caused by lattice mismatch, leading to TD bending in TD in the buffer layer^34^. These curved dislocations possess Burgers vectors of the same magnitude and opposite direction, which form pairs (Fig. 3(e,f)) and are eliminated near the interface. As a result, the TD propagation to the upper parts of the epilayer ceases. Figure 4(e) shows an HRTEM strain map along the vertical direction, produced by applying the Geometrical Phase Analysis (GPA) program on a dislocation loop with in the buffer layer, confirming the TD elimination near the interface. Furthermore, we observed TD defects grow vertically along the *c*-axis. Some of these defects stop propagating beyond the first 300 nm above the substrate, as shown in the HRTEM image (Fig. S1, supporting information) due to strain relaxation in the n-GaN epilayer above the distorted buffer layer^34^. This mechanism of low TDD is posited to be the reason behind the sharp (0002) RC peak of GaN grown on (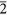
01)-oriented β-Ga_2_O_3_ with GaN buffer layer.

### Optical properties of n-GaN epilayer

**Figure 5:**
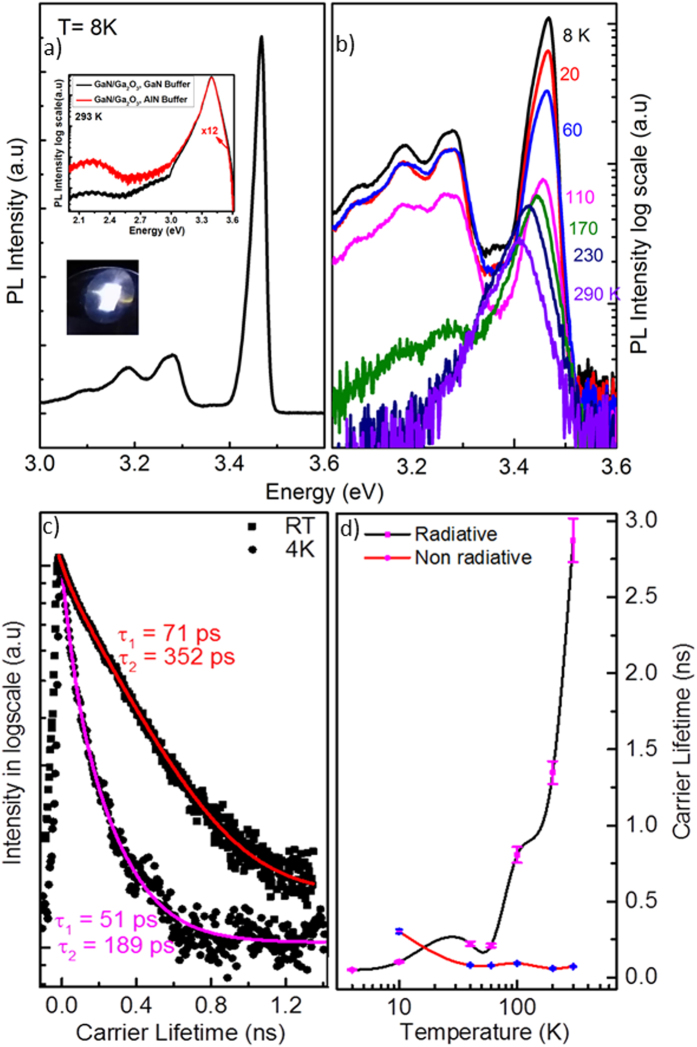
(**a**) PL spectra of n-GaN/(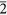
01)-oriented β-Ga_2_O_3_ epilayer at 8 K grown on a GaN buffer layer. The inset shows the RT PL spectra in comparison with a GaN epilayer grown on an AlN buffer layer. (**b**) Temperature dependence of the PL spectrum is measured between 8 K and RT. (**c**) Time-resolved PL spectra of the n-GaN epilayer measured at RT (squares) and at 4 K (circles) with the double exponential decay fit (red and magenta lines) for each temperature. (**d**) Temperature dependence of radiative recombination lifetimes (squares) and non-radiative recombination lifetimes (circles) taken from the sample.

A time-integrated low-temperature PL spectrum of the Si-doped GaN/(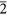
01)-oriented β-Ga_2_O_3_ epilayer (GaN buffer layer) is shown in Fig. 5(a). The spectrum displays an intense near-band-edge (NBE) emission under an excitation density of 6 W/cm^2^, centered at 3.47 eV, which has previously been attributed to band-to-band recombination and to bound and free exciton recombination^35^,^36^. The inset of Fig. 5(a) shows that a GaN epilayer grown on a GaN buffer layer rather than on an A1N buffer layer has a 12-fold higher PL intensity at RT with negligible yellow-band luminescence, suggesting a higher quality GaN epilayer and low TDD by using Low-temperature GaN buffer layer. The peak at 3.47 eV (Fig. 5(a)) can be attributed to direct transitions between the conduction and the valence band tail states, as well as random distribution of dopants, resulting in random fluctuations of the doping concentration on a microscopic scale^37^,^38^. At RT, a slight broadening in the FWHM of the NBE peak (74 meV) is observed, due to the presence of the Si dopant^39^. This broadening of the FWHM is expected as a result of high carrier concentration (at the low-energy side) introduced by Si impurities compared to undoped GaN and it can be explained by the tailing of the density of states caused by potential fluctuations introduced by the random distribution of Si dopants^39^. In addition, residual acceptors may contribute to the NBE peak broadening in the same way by introducing potential fluctuations^40^. Intensity power dependence studies show that decreasing the excitation intensity by three orders of magnitude produces no significant changes in the observed PL spectra, indicating that no band-gap filling or saturation of the defect level occurs and suggesting that these effects do not contribute to the broadening^41^. Peaks ascribed to donor-acceptor pair recombination accompanied by longitudinal optical (LO) phonon replicas were observed in the 3.28 − 2.98 eV energy range as shown in Fig. 5(a,b). The phonon replicas are observed in an energy difference of about 97 meV. Figure 5(b) shows a typical temperature-dependent PL spectrum of Si-doped GaN between 8 K and RT. As the temperature increases, the NBE peak weakens and becomes slightly red shifted. The LO phonon replicas weaken dramatically after 150 K and almost disappear at RT.

Figure 5(c) shows the TRPL spectra of Si-doped GaN/(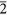
01)-oriented β-Ga_2_O_3_ epilayer (GaN buffer layer) at 4K and RT. The TRPL decay time of the Si-doped GaN epilayer can be described by a biexponential fitting. The biexponential decay process occurs in a multilevel system, which arises following the capturing of carriers at the deeper non-radiative centers either in the film or at the interface^42^,^43^. The biexponential decay can be described as^44^,


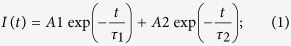


where A1 and A2 are adjustable constants, and *τ*_*1*_ and *τ*_*2*_ are the fast and slow decay times, respectively. Decay times for *τ*_*1*_ and *τ*_*2*_ are measured as 51 ps and 189 ps, respectively, at 4 K, and as 71 ps and 352 ps, respectively, at RT. The radiative recombination lifetime of the donor-bound exciton in intentionally and unintentionally doped GaN is typically in the range of 30 to 100 ps^45^ at RT, increasing to 530 ps at 4 K^41^. A high donor concentration (10^18^ cm^−1^) in a high-quality n-GaN epilayer has previously been shown to lead to a fast decay and a broad spectrum^43^. Hence, even at low-temperature, it will be difficult to clearly distinguish between free and donor-bound excitons. By defining the internal quantum efficiency η_int_(T) as the ratio of the time-integrated PL intensity at each temperature relative to that at the lowest temperature (4 K) and by assuming that the non-radiative channels are inactive at the lowest temperature (in line with the approach used in Rashba’s treatment^43^), we estimated the values of radiative τ_rad_(T) and non-radiative τ_nr_(T) recombination lifetimes using the following equations:


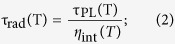



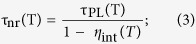


where τ_PL_(T) is the total recombination lifetime. The radiative and non-radiative recombination lifetimes obtained are plotted in Fig. 5(d). The squares and the circles indicate estimated τ_rad_ and τ_nr_, respectively. As the temperature increases from 4 K to RT, τ_rad_ increases super-linearly from 51ps to 2.8 ns as shown in Fig. 5(d). Such behavior has been reported at different Si-doping concentrations of GaN^46^. The decay time behavior of highly Si-doped GaN may be explained by the fact that the recombination is governed by bound excitons at low-temperatures (<10 K), whereas in the intermediate temperature range (20 to 100 K), the free exciton decay rate is largely weakened by the high electron background concentration (due to the presence of Si dopant atoms). Since the sample is heavily doped, the effect of the free excitons is weakened, and donor-to-band transitions significantly contribute to the recombination events at intermediate and higher temperatures (~100 to 300 K). However, for undoped- and moderately-doped GaN epilayers, the radiative decay time is considerably enhanced by the free exciton transition^43^. When temperature increases, the radiative lifetime decreases from 300 to 72 ps and non-radiative lifetime decreases, reflecting the thermal activation of non-radiative recombination processes (Fig. 5(d))^43^.

### The quality of InGaN grown on the n-GaN epilayer

**Figure 6:**
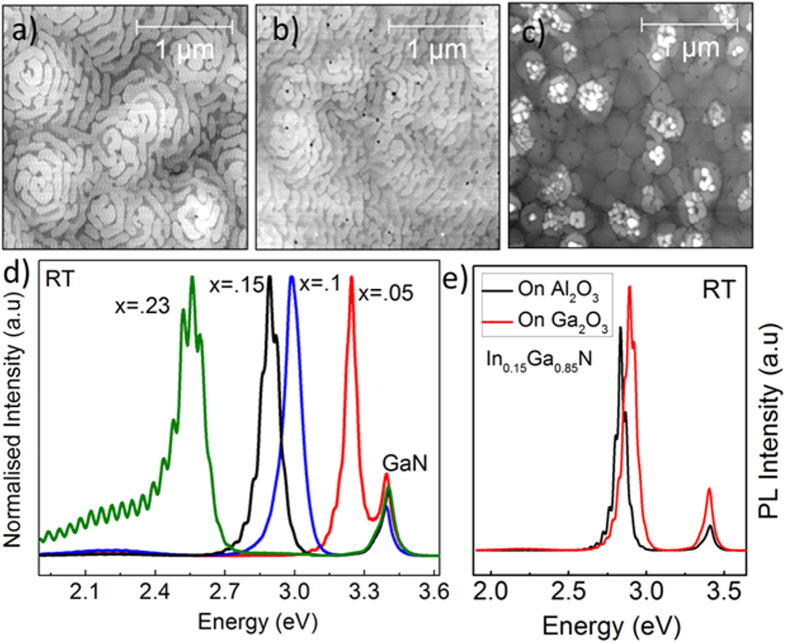
The 2.5 × 2.5-μm AFM images of the In_x_Ga_1−x_N epilayers for different. In concentrations (**a**) x = 0.1, (**b**) 0.15, and (**c**) 0.23. (**d**) The RT PL spectra of In_x_Ga_1−x_N films; spectra show oscillations due to interference at interfaces. (**e**) PL spectra comparison between In_0.15_Ga_0.85_N grown on Ga_2_O_3_ and Al_2_O_3_.

The growth of In_x_Ga_1−x_N epilayers by MOCVD is influenced by the surface morphology of the underlying n-GaN layer, growth mode, indium incorporation, and carrier gas composition^47^. It is, therefore, necessary to demonstrate that the Ga_2_O_3_ substrate can produce high structural and optical quality In_x_Ga_1−x_N materials grown on this n-GaN/(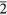
01)-oriented β-Ga_2_O_3_ (using GaN buffer layer) that can be used in the development of single and multiple quantum well vertical devices. We investigated the morphology of In_x_Ga_1−x_N samples through AFM (Fig. 6(a–c) whereby the surface roughness can be represented by the root-mean-square (RMS) average. Figure 6(a–c) show AFM images of In_0.1_Ga_0.9_N, In_0.15_Ga_0.85_N, and In_0.23_Ga_0.77_N thin films, respectively, deposited on n-GaN/β-Ga_2_O_3_. We observed low RMS roughness values of 0.91, 0.98, and 1.5 nm, for In_0.1_Ga_0.9_N, In_0.15_Ga_0.85_N, and In_0.23_Ga_0.77_N, respectively, indicating good quality smooth InGaN films on (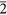
01)-β-Ga_2_O_3_ substrates using GaN buffer layer, compared to Al_2_O_3_ and Si substates^48^. At least one pit (dislocation) located in the center of each spiral is observed for all InGaN epilayers, as shown in Fig. 6(a–c). Figure 6(d) shows the normalized PL spectra of these In_x_Ga_1−x_N thin films, which exhibit an intense NBE emission at RT. The PL NBE peaks arising from In_0.05_Ga_0.95_N, In_0.1_Ga_0.9_N, In_0.15_Ga_0.85_N, and In_0.23_Ga_0.77_N were observed at 383, 415, 428, and 487 nm, respectively. Moreover, the PL FWHM of these In_x_Ga_1−x_N NBE peaks increases from 60 to 110 nm with increasing In concentration. The observed FWHM broadening is attributed to carrier localization due to the inhomogeneous compositional fluctuation of InN across the film as In concentration increases^49^. Furthermore, PL emissions of all In_x_Ga_1−x_N films grown on the (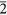
01) β-Ga_2_O_3_ substrate showed high peak intensity compared to those grown on Al_2_O_3_ substrates (Fig. 6(e)), indicating that the β-Ga_2_O_3_ substrate is a potential candidate for growing good quality InGaN materials. The blueshift of NBE peak (Fig. 6(e)) of the InGaN sample grown on (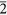
01) β-Ga_2_O_3_ compared to that grown on Al_2_O_3_ can be due to a different degree of strain and InN compositional fluctuation.

## Conclusion

We have reported high optical and structural quality In_x_Ga_1−x_N epilayers (0 ≤ x ≤ 23) grown on (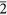
01) β-Ga_2_O_3_ substrate using low-temperature undoped GaN buffer layer β-Ga_2_O_3_ is a potential substrate material for III-nitrides, which incorporates the transparency of sapphire and the conductivity of SiC. Raman mapping and XRD RC show high-quality uniform and nearly strain-free 2″ wafer grown on (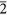
01) β-Ga_2_O_3_ substrate by using GaN buffer layer, confirming that this substrate can be used for large-scale technology for nitride-based vertical devices. TEM analysis confirms the low TDD of the single-crystalline epilayers. We found that the TD annihilation occurred near the interface between the substrate and the buffer layer, as a result of TDs bending into the basal plane and reacting with dislocations of the opposite phase, before eliminating each other. The TD bending can be ascribed to the combination of the nature of the (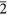
01) β-Ga_2_O_3_ surface (nano-humps) and the buffer layer distortion, followed by roughly strain-free single crystal Si-doped GaN epilayer, preventing these TD continuing propagating to this epilayer. The PL measurements revealed a high-intensity NBE emission peak with a weak yellow band, whereas TRPL showed a low non-radiative contribution to the NBE emission. This work demonstrates that improved quality and stable III-nitride crystals grown on (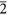
01)-β-Ga_2_O_3_ substrate can be obtained, relative to those grown on (100)-orientated β-Ga_2_O_3_. We showed that conductive β-Ga_2_O_3_ promises better current distribution, thermal management and better light extraction compared to sapphire and testifies to its suitability as a substrate for manufacturing large scale high-efficiency vertical devices, which increases the lifetime and simplifies the fabrication process compared to lateral devices. Dislocation density in nitride films grown on (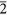
01)-oriented β-Ga_2_O_3_ may be further reduced as crystal growth continues to be optimized either by lateral overgrowth or substrate patterning.

## Additional Information

**How to cite this article**: Muhammed, M. M. *et al*. High-quality III-Nitride films on conductive, transparent (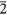
01)-oriented β-Ga_2_O_3_ using a GaN buffer layer. *Sci. Rep*. **6**, 29747; doi: 10.1038/srep29747 (2016).

## Supplementary Material

Supplementary Information
