# Supplementary material for: High-quality III-nitride films on conductive, transparent (2̅01)-oriented β-Ga2O3 using a GaN buffer layer
Source: Sci Rep. 2016 Jul 14;6:29747. doi: 10.1038/srep29747 (PMC4944183; doi:10.1038/srep29747)
Supplement: Supplementary Information [file srep29747-s1.pdf]

Supporting Information

High-quality III-nitride films on conductive, transparent $(\bar{2}01)$ β-Ga_2O_3 using a GaN buffer layer

M. M. Muhammed,[1] M. A. Roldan,[2] Y. Yamashita,[4] S.-L. Sahonta,[3] I. A. Ajia,[1] K. Iizuka,[4] A. Kuramata,[4] C.J. Humphreys,[3] and I. S. Roqan[1*]

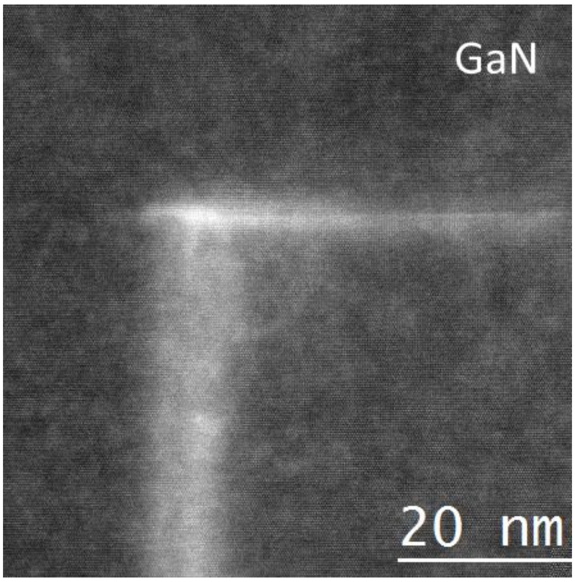

Figure S1. Cross-sectional HRTEM images showing TD defects grow vertically along the *c*-axis and stop propagating beyond the first 300 nm above the substrate.

* Corresponding author iman.roqan@kaust.edu.sa
